# A Systematic Review and Meta-Analysis Comparing the Efficacy of Enteral Antibiotics Combined With Mechanical Bowel Preparation Versus Mechanical Bowel Preparation Alone in Elective Colorectal Resections

**DOI:** 10.7759/cureus.87991

**Published:** 2025-07-15

**Authors:** Anurag Singh, Ehtisham Zeb, Haitham Tumeh, Mostafa Abdel-Halim, Mamoon Solkar

**Affiliations:** 1 Colorectal Surgery, Tameside and Glossop Integrated Care NHS Foundation Trust, Manchester, GBR

**Keywords:** colorectal cancer, colorectal cancer surgery, colorectal resections, large bowel, mechanical bowel preparation (mbp)

## Abstract

This study compares the outcomes of elective colorectal surgery in patients who received enteral antibiotics (EAb) in addition to standard care (SC) (mechanical bowel preparation and intravenous antibiotics) to those who received SC alone. This study will review existing research on the impact of prophylactic EAb on colorectal surgery outcomes. Relevant studies were identified from electronic medical databases and analysed using the statistical software RevMan (Review Manager 5.4, The Nordic Cochrane Centre, Copenhagen, Denmark) based on the principles of meta-analysis. A total of 13 studies (nine RCTs and four comparative trials) on 9,466 patients for postoperative outcomes were included. A random-effects model analysis was conducted, revealing that anastomotic leak rates were higher in the control group, with no heterogeneity observed. Surgical site infections were higher in the control group with moderate heterogeneity. Postoperative mortality and readmission rates were similar in both groups. The use of EAb is associated with a reduced incidence of anastomotic leaks and surgical site infections; however, readmission rates and mortality require further assessment in a major, multicenter, randomised study.

## Introduction and background

Colorectal cancer is the second most common cause of cancer-related diseases globally [1]. It remains a serious global burden, with approximately 1.8 million cases diagnosed in 2018 [2]. In the UK alone, colorectal cancer is the fourth most common cause of cancer, with over 42,000 patients being diagnosed every year [3]. Surgery remains the mainstay of management for colorectal cancer patients [4].

The most problematic complications after colorectal surgery remain the anastomotic leak (AL) and surgical site infections (SSIs), with incidence ranging from 3% to 30% [5,6]. These complications are associated with high morbidity and mortality for these patients, with mortality from AL going up to 1.7%-16.4% [7,8]. Several factors have been identified as contributing to these complications, including surgery-related factors, the surgeon's technique, mechanical bowel preparation (MBP), or antibiotic use [9], as well as patient-related factors such as smoking and obesity [10,11].

In general, antibiotic prophylaxis along with MBP has been shown to reduce the risk of SSIs and AL [12]. Globally, some surgeons prefer to use a combination of oral and intravenous antibiotics (IV Ab) [13], while others in Europe and Asia prefer to use IV Ab alone [14,15]. Multiple studies have examined the use of EAb in combination with IV Ab, revealing conflicting results [16-20].

This systematic review and meta-analysis aims to understand the effects of enteral antibiotics (EAb) on patients undergoing elective colorectal surgery and compare the postoperative outcomes with those of patients without EAb.

The abstract of this study was presented at the Association of Surgeons in Training annual conference 2025 in Belfast, UK.

## Review

Methods

Data Sources and Literature Search Techniques

The electronic databases PubMed, EMBASE, MEDLINE, and the Cochrane Library were carefully searched and scrutinised. Appropriate articles were identified after formulating a search strategy, and MeSH (Medical Subject Headings) terms (colorectal surgery, enteral antibiotic prophylaxis, mechanical bowel preparation, and colorectal resection) were used along with the Boolean operator (AND) to refine and narrow down the search results identified with this strategy. References of the articles identified were further analysed to identify any noteworthy, published articles appropriate for the research question.

Trial Selection

This systematic review and meta-analysis included studies that compared enteral antibiotics combined with mechanical bowel preparation (EAMBP) to MBP alone before colorectal surgery. Postoperative outcomes were compared in these studies.

Data Collection and Management

The authors identified and extracted data from eligible studies, and the data were stored in a standardised electronic format. The data collected was analysed by the authors, ensuring that mutual agreement was reached. Extracted data included study title, study design, year of publication, intervention type (EAMBP or MBP alone), demographic characteristics of the study population, and postoperative complications, including AL and SSI.

Evidence Synthesis Using RevMan Statistical Software

RevMan version 5.4 (Review Manager 5.4, The Nordic Cochrane Centre, Copenhagen, Denmark) was used for the statistical analysis of the data [20]. The risk ratio (RR) and standardised mean difference (SMD) with a 95% confidence interval (CI) were used to present the summated outcome of binary and continuous variables, including AL, SSIs, 30-day mortality, and readmission rate. The SMD and RR were calculated and presented with a 95% CI under the random-effects model analysis [21,22]. A forest plot was used for the graphical presentation of the results. The statistical heterogeneity was calculated using the chi-squared test, with a significance level set at P < 0.05. The quantification of heterogeneity was assessed using the I² test, with a maximum value of 30% indicating low heterogeneity [23]. For the calculation of the SMD, the inverse-variance method was used. For the calculation of the RR, the Mantel-Haenszel method was employed under a random-effects model analysis [24,25]. If standard deviation was not reported in the published article on randomised controlled trials (RCTs), it was estimated either from the range or p-value, or 0.5 was added to the cell frequency, assuming the same variance in both groups, which might not be true in all cases. The estimate of the difference between the two techniques was pooled, depending on the effect weights in the results, which were determined by each trial's estimated variance.

Outcomes

The primary outcome of this systematic review and meta-analysis was postoperative AL and SSIs. The secondary outcomes included readmission rates and 30-day mortality rates.

Results

**Figure 1:**
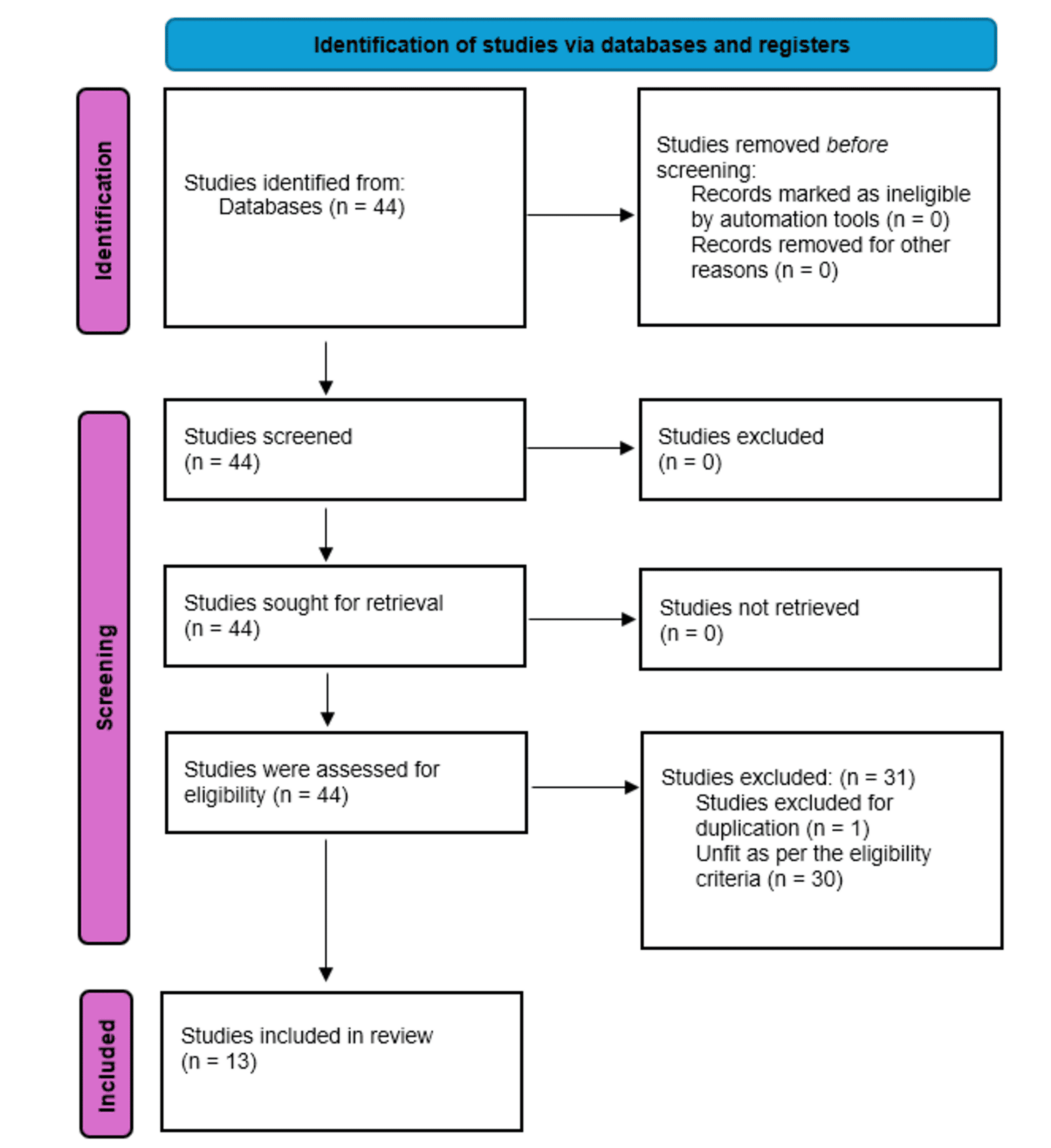
PRISMA flowchart showing literature search outcomes. PRISMA: Preferred Reporting Items for Systematic Reviews and Meta-Analyses

The primary search, conducted using the specified search strategy, yielded 44 studies. After going through the different stages of the screening, 13 studies were identified, out of which nine studies were RCTs and four were comparative trials. Figure 1 shows the search strategy in the PRISMA (Preferred Reporting Items for Systematic Reviews and Meta-Analyses) flowchart.

Characteristics and Demographics of Included Studies

Nine RCTs [26-34] and four comparative trials [35-38] with a total of 9,466 patients were included in the study of postoperative outcomes. Two studies were reported from the Netherlands [26,37], one study from China [27], one study from Greece [28], five studies from Japan [29-30,32-34], one study from the Republic of Korea [35], two studies from the UK [36,38], and one study from Germany [31]. Table 1 presents the characteristics of the included RCTs, and Table 2 displays the treatment protocols used in the included studies.

**Table 1:** Demographics of the included studies * Calculated indirectly RCT: randomised controlled trial; NR: not reported; SD: standard deviation

Study	Country	Type	Age (Mean ± SD) (Years)		Gender (Female %)	
			Enteral Abx	No enteral Abx	Enteral Abx	No enteral Abx
Abis et al., 2019 [26]	Netherlands	RCT	67.5 ± 8.4	68.1 ± 9.0	42.5	40.96
Anjum et al., 2017 [27]	China	RCT	46.3 ± 14.4	45.2 ± 15.6	35.78	37.78
Frountzas et al., 2024 [28]	Greece	RCT	70 ± 11*	70 ± 11*	42*	42*
Hata et al., 2016 [29]	Japan	RCT	67.5 ± 8.4	68.1 ± 9.0	39.7	47.1
Ikeda et al., 2016 [30]	Japan	RCT	60 ± 16.5	57.5 ± 14.25	44.53	44.92
Ishida et al., 2001 [33]	Japan	RCT	62 ± 12.5	55 ± 17	34.72	40.84
Kobayashi et al., 2007 [34]	Japan	RCT	67.9 ± 15.25*	69.1 ± 12.25*	43.38	36.36
Lee et al., 2021 [35]	Republic of Korea	Comparative	64.7 ± 11.8	65.7 ± 11.9	40.1	40.1
Najdawi et al., 2021 [36]	UK	Comparative	NR	NR	36.4	51.6
Roos et al., 2009 [37]	Netherlands	Comparative	64 ± 15	63 ± 15	57	52
Schardey et al., 2020 [31]	Germany	RCT	61 ± 7.5	64 ± 6.5	27	40
Takesue et al., 2000 [32]	Japan	RCT	68 ± 14	65 ± 11.75	34.21	35.55
Vadhwana et al., 2020 [38]	UK	Comparative	68.7 ± 13.6	68.3 ± 13.2	60.6	55.1

**Table 2:** Treatment protocol among the included studies NR: not reported; MBP: mechanical bowel preparation; IV: intravenous; GI: gastrointestinal

Study	Enteral Antibiotics Group	No Enteral Antibiotics Group
			Abis et al., 2019 [26]	Diagnosis: biopsy-proven colorectal cancer	Diagnosis: biopsy-proven colorectal cancer
Surgery: elective surgery, primary anastomosis, laparoscopic or open surgery	Surgery: elective surgery, primary anastomosis, laparoscopic or open Surgery		
Enteral antibiotics: 1 g Amphotericin B + 100 mg Colistin + 80 mg Tobramycin	IV antibiotics: 1 g Cefazolin + 500 mg Metronidazole		
IV antibiotics: 1 g Cefazolin + 500 mg Metronidazole	MBP: for left-sided resections		
MBP: for left-sided resections			
Anjum et al., 2017 [27]	Diagnosis: GI fistula, IBD, trauma, malignancy	Diagnosis: GI Fistula, IBD, Trauma, Malignancy
	Surgery: elective surgery, laparoscopic or open surgery	Surgery: elective surgery, laparoscopic or open surgery
	Enteral antibiotics: Metronidazole 400 mg + levofloxacin 200 mg (3 doses)	IV antibiotics: Cephalosporin and Metronidazole
	IV antibiotics: Cephalosporin and Metronidazole	MBP: sodium phosphate
	MBP: sodium phosphate	
Frountzas et al., 2024 [28]	Diagnosis: colorectal cancer	Diagnosis: colorectal cancer
	Surgery: elective open surgery	Surgery: elective open surgery
	Oral antibiotics: 3 doses of 400 mg rifaximin + 1 dose of 500 mg metronidazole	IV Antibiotics: 1 dose of 2 g cefoxitin + 500 mg metronidazole
	IV antibiotics: 1 dose of 2 g cefoxitin + 500 mg metronidazole	MBP: 2 doses of oral sodium phosphate
	MBP: 2 doses of oral sodium phosphate	
Hata et al., 2016 [29]	Diagnosis: colorectal cancer or adenoma	Diagnosis: colorectal cancer or adenoma
	Surgery: elective laparoscopic colorectal surgery	Surgery: elective laparoscopic colorectal surgery
	Oral antibiotics: 2 doses of 1 g kanamycin + 750 mg metronidazole	IV antibiotics: 1 g cefmetazole
	IV antibiotics: 1 g cefmetazole	MBP: 75 mg sodium picosulphate + 34 mg magnesium citrate
	MBP: 75 mg sodium picosulphate + 34 mg magnesium citrate	
Ikeda et al., 2016 [30]	Diagnosis: colorectal cancer	Diagnosis: colorectal cancer
	Surgery: elective laparoscopic colorectal resection	Surgery: elective laparoscopic colorectal resection
	Oral antibiotics: 2 doses of 1 g kanamycin + 750 mg metronidazole	IV Antibiotics: 1 g cefmetazole
	IV antibiotics: 1 g cefmetazole	MBP: sodium picosulphate + Magnesium citrate
	MBP: sodium picosulphate + magnesium citrate	
Ishida et al., 2001 [33]	Diagnosis: colorectal cancer, IBD, diverticular disease, others	Diagnosis: colorectal cancer, IBD, diverticular disease, others
	Surgery: colectomy (32), anterior resections (29), others (11)	Surgery: colectomy (44), anterior resections (18), others (9)
	Oral antibiotics: Kanamycin 2 g/day and Erythromycin 1.6 g/day	IV antibiotics: 1 g Cefotaxime at induction
	IV Antibiotics: 1 g Cefotaxime at induction	MBP: polyethylene glycol
	MBP: polyethylene glycol	
Kobayashi et al., 2007 [34]	Diagnosis: colorectal cancer	Diagnosis: colorectal cancer
	Surgery: colon (120), rectum (122)	Surgery: colon (121), rectum (121)
	IV antibiotics: 1 g Cefotaxime at induction	IV antibiotics: 1 g Cefotaxime at induction
	Oral Antibiotics: Kanamycin 1 g and Erythromycin 400 mg, 3 doses	MBP: polyethylene glycol
	MBP: polyethylene glycol	
Lee et al., 2021 [35]	Diagnosis: colorectal cancer	Diagnosis: colorectal cancer
	Surgery: laparoscopic/open colorectal surgery	Surgery: laparoscopic/open colorectal surgery
	Oral antibiotics: Metronidazole/Quinolone/Macrolide/Rifaximin: 1 day	IV antibiotics: Cephalosporins
	IV Antibiotics: cephalosporins	MBP: polyethylene glycol or sodium picosulphate
	MBP: polyethylene glycol or sodium picosulphate	
Najdawi et al., 2021 [36]	Diagnosis: colorectal cancer	Diagnosis: colorectal cancer
	Surgery: laparoscopic colorectal resection	Surgery: laparoscopic colorectal resection
	Oral antibiotics: 7-day pre-op neomycin	IV antibiotics: NR
	IV antibiotics: NR	MBP: NR
	MBP: NR	
Roos et al., 2009 [37]	Diagnosis: NR	Diagnosis: NR
	Surgery: all colorectal resections	Surgery: all colorectal resections
	Oral antibiotics: polymyxin B sulphate 100 mg/Tobramycin 80 mg/Amphotericin B 500 mg	IV antibiotics: Cefuroxim 1.5 g + Metronidazole 500 mg
	IV Antibiotics: Cefuroxim 1.5 g + Metronidazole 500mg	MBP: Klean-prep (osmotic laxative)
	MBP: Klean-prep (osmotic laxative)	
Schardey et al., 2020 [31]	Diagnosis: colorectal cancer	Diagnosis: colorectal cancer
	Surgery: low anterior resection	Surgery: low anterior resection
	Oral antibiotics: Polymyxin B 100 mg + Tobramycin 80 mg + Vancomycin 125 mg + Amphotericin B 500 mg four daily doses, started one day preoperatively until day seven postoperatively	IV antibiotics: Amphotericin B 500 mg
	IV Antibiotics: Amphotericin B 500 mg	MBP: Klean-prep (osmotic laxative)
	MBP: Klean-prep (osmotic laxative)	
Takesue et al., 2000 [32]	Diagnosis: NR	Diagnosis: NR
	Surgery: open colorectal resections	Surgery: open colorectal resections
	Oral antibiotics: Kanamycin 500 mg + Metronidazole 500 mg, 3 doses	IV Antibiotics: Cefmetazole 1 g at induction
	IV Antibiotics: Cefmetazole 1 g at induction	MBP: polyethylene glycol
	MBP: polyethylene glycol	
Vadhwana et al., 2020 [38]	Diagnosis: NR	Diagnosis: NR
	Surgery: open/laparoscopic colorectal resections	Surgery: open/laparoscopic colorectal resections
	Oral antibiotics: Neomycin/Erythromycin/Metronidazole	IV antibiotics: Gentamycin + Metronidazole/Co-amoxiclav
	IV antibiotics: Gentamycin + Metronidazole/Co-amoxiclav	MBP: Klean-prep (osmotic laxative)
	MBP: Klean-prep (osmotic laxative)	

Methodological Quality of Included Studies

The reported quality variables in the included RCTs, used to assess the strength of the published evidence, are summarised in Table 3. The randomisation technique used in the included RCTs and comparative trials was performed according to the Cochrane Collaboration tool [39-41].

**Table 3:** Quality of the included randomised controlled trials NR: not reported

Study	Randomisation Technique	Concealment	Blinding	Intention-to-Treat Analysis	Ethical Approval	Registration Number	Power Calculation
Abis et al., 2019 [26]	Computer-generated	Online	NR	Reported	Reported	NCT01740947	Reported
Anjum et al., 2017 [27]	Computer-generated	NR	Double	Reported	Reported	NR	Reported
Frountzas et al., 2024 [28]	Block randomisation	NR	Double	NR	Reported	NCT03563586	Reported
Hata et al., 2016 [29]	Computer-generated	Nil	Nil	Reported	Reported	NCT00508690	Reported
Ikeda et al., 2016 [30]	Sequentially numbered	Sealed envelopes	NR	NR	Reported	UMIN000019339	Reported
Ishida et al., 2001 [33]	Manual randomisation	Sealed envelopes	Single	NR	NR	NR	NR
Kobayashi et al., 2007 [34]	NR	NR	NR	Reported	Reported	NR	Reported
Schardey et al., 2020 [31]	NR	Double	NR	Reported	Reported	NR	NR
Takesue et al., 2000 [32]	NR	NR	NR	NR	NR	NR	NR

Primary Outcomes

**Figure 2:**
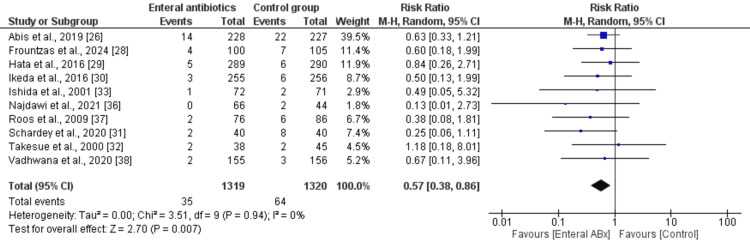
Forest plot showing the anastomotic leak rate in elective colorectal resection between the enteral Abx group and the control group. The outcome is presented as a risk ratio with a 95% confidence interval.

In the random-effects model analysis, the incidence of AL was higher in the MBP group when compared with the EAMBP group (RR 0.57, 95% CI (0.37, 0.86), Z = 2.67, P = 0.008) (Figure 2). There was no heterogeneity among the included studies (Tau² = 0.00; Chi² = 3.48, df = 8; P = 0.90; I² = 0%).

**Figure 3:**
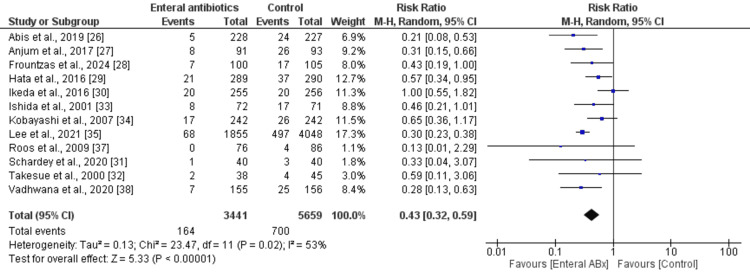
Forest plot showing surgical site infections in elective colorectal resection between the enteral Abx group and the control group. The outcome is presented as a risk ratio with a 95% confidence interval.

In the random-effects model analysis, the incidence of SSIs was higher in the MBP group compared with the EAMBP group (RR 0.47, 95% CI (0.32, 0.67), Z = 4.06, P = 0.001) (Figure 3). There was a moderate heterogeneity among the included studies (Tau² = 0.17; Chi² = 22.48, df = 9; P = 0.007; I² = 60%).

Secondary Outcomes

**Figure 4:**
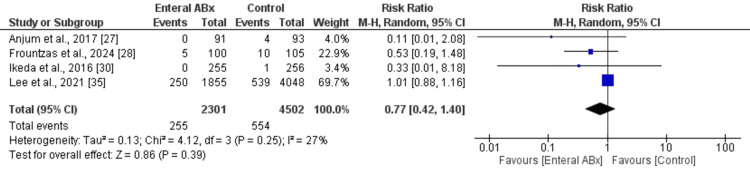
Forest plot showing the readmission rate in elective colorectal resection between the enteral Abx group and the control group. The outcome is presented as a risk ratio with a 95% confidence interval.

In the random-effects model analysis, the incidence of 30-day mortality was statistically similar in both groups (RR 0.71, 95% CI (0.34, 1.48), Z = 0.92, P = 0.36) (Figure 4). There was no heterogeneity among the included studies (Tau² = 0.00; Chi² = 0.01, df = 1; (P = 0.93; I² = 0%).

**Figure 5:**
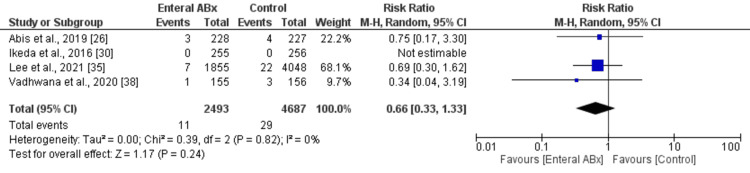
Forest plot showing the 30-day mortality in elective colorectal resection between the enteral Abx group and the control group. The outcome is presented as a risk ratio with a 95% confidence interval.

In the random-effects model analysis, the incidence of readmission was statistically similar in both groups (RR 1.00, 95% CI (0.87, 1.15), Z = 0.02, P = 0.98) (Figure 5). There was no heterogeneity among the included studies (Tau² = 0.00; Chi² = 1.96, df = 2; P = 0.38; I² = 0%).

Discussion

This systematic review and meta-analysis provides compelling evidence that preoperative EAb, when administered alongside MBP, significantly reduces the incidence of AL and SSIs in patients undergoing colorectal surgery compared to a control group without antibiotics. These primary endpoints demonstrate clear superiority in the intervention group, suggesting that EAb play a critical role in minimising infection-related complications. The findings are particularly notable given the established link between anaerobic bacteria in the colon and SSIs following colorectal procedures. By reducing the bacterial load prior to surgery, EAb appears to address a key pathophysiological driver of these complications. In contrast, the secondary outcomes (30-day mortality and readmission rates) showed no statistically significant difference between the intervention and control groups. This suggests that while EAb may effectively impact local surgical outcomes, its influence on broader postoperative morbidity and mortality remains limited, warranting further investigation.

The observed reduction in SSIs with preoperative EAb aligns with a growing body of literature. Multiple studies [26,29,42,43] have previously demonstrated similar benefits, reinforcing the biological plausibility of this approach. For instance, a systematic review by Grewal et al. [44] reported comparable outcomes in patients undergoing colorectal resections for cancer, where selective decontamination of the digestive tract with oral antibiotics led to decreased infection rates. Similar results with selective decontamination have also been observed in the SELECT trial [45]. These consistent findings across studies suggest that preoperative EAb could be a valuable component of perioperative care, particularly in high-risk procedures such as colorectal surgery. However, the lack of difference in secondary outcomes, such as mortality and readmission rates, raises questions about the broader clinical impact of this intervention. It is possible that while EAb mitigates immediate surgical complications, other factors, such as patient comorbidities, the quality of postoperative care, or systemic inflammatory responses, may overshadow its effects on longer-term outcomes.

The use of preoperative EAb, particularly in combination with standard care (SC) such as MBP, has been a topic of debate for decades, and this study highlights the ongoing uncertainty in clinical practice. A survey of the American Society of Colon and Rectal Surgeons [46] revealed a striking diversity of opinions: approximately 10% of respondents deemed preoperative oral antibiotic prophylaxis unnecessary and advocated for its discontinuation, 50% endorsed its routine use for all colorectal surgery patients, and 40% remained undecided. This lack of consensus likely stems from variability in the evidence base, including differences in study design, antibiotic regimens, and patient populations. The present meta-analysis, being the most extensive to date, makes a robust contribution to this discourse by synthesising data from multiple trials. Nevertheless, its findings must be interpreted in light of its limitations, which may perpetuate some of the confusion among clinicians.

Several limitations of this systematic review warrant careful consideration when evaluating its implications. First, there was significant heterogeneity in the choice of preoperative EAb across the included studies. Agents, dosages, and administration protocols varied, potentially influencing efficacy and complicating efforts to identify an optimal regimen. Second, the patient population was diverse, encompassing diagnoses such as colorectal cancer, inflammatory bowel disease (IBD), diverticular disease, and others. These conditions differ in their underlying pathophysiology, surgical complexity, and microbial profiles, which could introduce variability in treatment response and outcomes. For example, the inflamed gut in IBD patients may respond differently to antibiotics compared to the relatively stable colon in early-stage colorectal cancer. Third, the quality of the included RCTs was inconsistent, with some studies exhibiting methodological weaknesses such as inadequate blinding or small sample sizes. This non-uniformity reduces the overall strength of the evidence and underscores the need for caution when generalising the results.

Despite these limitations, this study provides the most comprehensive evidence to date supporting the use of preoperative EAb to reduce AL and SSIs in colorectal surgery. The observed benefits are clinically meaningful, as both complications are associated with significant morbidity, prolonged hospital stays, and increased healthcare costs. However, several knowledge gaps remain. The ideal antibiotic agent, its optimal dosage, and the recommended duration of administration have yet to be definitively established. For instance, should broad-spectrum antibiotics targeting anaerobes be prioritised, or could narrower-spectrum agents suffice? Additionally, the timing of administration, whether a single dose or a multi-day course, requires further investigation to balance efficacy with the risk of antibiotic resistance. Moreover, the lack of impact on secondary outcomes, such as 30-day mortality and readmission rates, suggests that future research should explore additional parameters, including postoperative ileus, length of hospital stay, or quality of life, to provide a more comprehensive assessment of EAb's utility.

Beyond these immediate questions, the findings invite broader reflection on the role of the gut microbiome in surgical outcomes. The reduction in anaerobic bacteria linked to lower SSI rates hints at a mechanistic pathway that could be further elucidated through microbiome sequencing studies. Such research might identify specific bacterial taxa most responsible for complications, potentially guiding the development of targeted antibiotic or probiotic strategies. Additionally, the heterogeneity in patient diagnoses raises the possibility of personalised approaches: could certain subgroups (e.g., cancer patients versus IBD patients) benefit more from tailored regimens? Addressing these questions could refine the application of EAb and enhance its integration into clinical guidelines.

The implications for practice are significant but tempered by the need for additional evidence. While this meta-analysis strengthens the case for preoperative EAb, the variability in current practice and the study's limitations suggest that universal adoption may be premature. Instead, clinicians might consider EAb as a standard option for high-risk patients, such as those undergoing complex resections or with a history of prior infections, while awaiting more definitive data. Updating surgical guidelines to reflect these findings could be a reasonable step, provided recommendations remain flexible to accommodate ongoing research.

## Conclusions

In conclusion, this systematic review and meta-analysis offers robust evidence that preoperative EAb, combined with MBP, reduces AL and SSIs in colorectal surgery, affirming their role in improving surgical outcomes. However, the heterogeneity in antibiotic use, patient populations, and trial quality, coupled with the lack of effect on mortality and readmission, highlights the need for further investigation. Future studies should prioritise standardising antibiotic protocols, exploring microbiome dynamics, and assessing a wider range of postoperative outcomes to fully delineate the benefits and limitations of this approach. Until then, this study stands as a critical milestone in the ongoing effort to optimise perioperative care in colorectal surgery.
